# Silver nanoparticles loaded on lactose/alginate: in situ synthesis, catalytic degradation, and pH-dependent antibacterial activity

**DOI:** 10.3762/bjnano.14.64

**Published:** 2023-07-04

**Authors:** Nguyen Thi Thanh Tu, T Lan-Anh Vo, T Thu-Trang Ho, Kim-Phuong T Dang, Van-Dung Le, Phan Nhat Minh, Chi-Hien Dang, Vinh-Thien Tran, Van-Su Dang, Tran Thi Kim Chi, Hieu Vu-Quang, Radek Fajgar, Thi-Lan-Huong Nguyen, Van-Dat Doan, Thanh-Danh Nguyen

**Affiliations:** 1 Faculty of Applied Technology, School of Technology, Van Lang University, Ho Chi Minh City, Vietnam; 10 Faculty of Chemical Engineering, Industrial University of Ho Chi Minh City, Ho Chi Minh City, Vietnam; 2 Institute of Chemical Technology, Vietnam Academy of Science and Technology, 1A TL29 Street, District 12, Ho Chi Minh City 700000, Vietnam; 3 Graduate University of Science and Technology, Vietnam Academy of Science and Technology, 18 Hoang Quoc Viet, Cau Giay, Hanoi 11000, Vietnam; 4 Faculty of Environment Ho Chi Minh City University of Natural Resources and Environment, 236B Le Van Sy Street, Tan Binh District, Ho Chi Minh City 700000, Vietnam; 5 Department of Chemical Technology, Ho Chi Minh City University of Food Industry, Ho Chi Minh City 700000, Vietnam; 6 Institute of Materials Science, Vietnam Academy of Science and Technology, 18 Hoang Quoc 14 Viet, Cau Giay District, Hanoi 11000, Vietnam; 7 NTT Hi-Tech Institute, Nguyen Tat Thanh University, Ho Chi Minh City 700000, Vietnam; 8 Institute of Chemical Process Fundamentals of the AS CR Prague, Czech Republic; 9 Institute of Biotechnology and Food Technology, Industrial University of Ho Chi Minh City, Ho Chi Minh City, Vietnam

**Keywords:** alginate, bacterial activity, catalysis, lactose, silver nanoparticles, synthesis

## Abstract

We present the in situ synthesis of silver nanoparticles (AgNPs) through ionotropic gelation utilizing the biodegradable saccharides lactose (Lac) and alginate (Alg). The lactose reduced silver ions to form AgNPs. The crystallite structure of the nanocomposite AgNPs@Lac/Alg, with a mean size of 4–6 nm, was confirmed by analytical techniques. The nanocomposite exhibited high catalytic performance in degrading the pollutants methyl orange and rhodamine B. The antibacterial activity of the nanocomposite is pH-dependent, related to the alterations in surface properties of the nanocomposite at different pH values. At pH 6, the nanocomposite demonstrated the highest antibacterial activity. These findings suggest that this nanocomposite has the potential to be tailored for specific applications in environmental and medicinal treatments, making it a highly promising material.

## Introduction

Silver nanoparticles (AgNPs) have raised significant interest for their wide range of applications in biomedicine [1–2], treatment of wastewater [3–4], and catalysis [5–6]. The utilization of eco-friendly sources, such as plant extracts [7–8], fungi [9–10], and bacteria [11], for synthesizing AgNPs is an important topic in the field of nanotechnology. However, AgNPs synthesized in that way have a number of drawbacks such as high cost and low stability. Typically, AgNPs are loaded onto polymers/composites in order to enhance their performance in usage [4,10,12]. Among polymers, polysaccharides have shown significant improvement in stabilization because of the hydroxy groups in the sugar molecules of the polysaccharide chain, which can form strong associations with AgNPs [13–15]. Previous studies have reported that metallic nanoparticles can be easily incorporated into polysaccharides by an in situ reduction process that requires no additional chemicals [16–17]. This technique using polysaccharides such as alginate [18] or chitosan [19] in conjunction with reducing agents enhanced cost efficiency and reduced the amounts of impurities or toxic compounds.

AgNPs are widely used as antibacterial agents. One of the advantages of AgNPs over bulk metal or salts is their ability to controllably release silver as ions or particles, resulting in prolonged protection against bacteria [20]. While the specific mechanism of their antibacterial properties is not fully understood, mechanisms such as release of silver ions or electrostatic interaction between AgNPs and microbial cells, have been proposed [21–22]. The AgNPs might release silver ions capable of binding to nucleic acids, thereby, exhibiting antibacterial activity [23–24]. Consequently, any silver-containing composite material with antibacterial properties can serve as a source of silver ions. Another mechanism involves the electrostatic attraction between negatively charged microbial cells and positively charged AgNPs [25]. Because of their affinity to sulfur proteins and through electrostatic attraction, silver ions can bind to both cytoplasm and cell wall, leading to increased permeability and, eventually, disruption of the bacterial cell wall [26]. This disruption can deactivate respiratory enzymes, resulting in the generation of reactive oxygen species [27] and the inhibition of adenosine triphosphate release [26]. The antibacterial properties of AgNPs can be influenced by factors such as size, pH value, and ionic strength of the medium [28–31]. Extensive research has been dedicated to control the size and shape of AgNPs through the application of various capping agents. In addition to their antimicrobial properties, AgNPs are also valuable catalysts for the removal of environmental contaminants in aqueous solutions. The high surface-to-volume ratio of AgNPs provides many active sites, thereby, enhancing their catalytic activity [32]. The catalytic activity of AgNPs is also influenced by the morphology and the use of capping agents [33]. The efficiency of the catalyst can be improved by studying different carrier materials [34–35]. Interface factors, including morphology and capping agents, play a significant role in the catalytic activity of AgNPs. The ionotropic gelation mechanism has been recently employed to create nanocomposites using saccharides, such as alginate, cyclodextrin, and lactose. This method allows for the loading of metallic ions onto the nanocomposite, followed by their reduction to nanoparticles [36–37]. The nanocomposite matrix can release silver in appropriate environments, exhibiting pH-dependent antibacterial activity [38].

In this study, we prepared a nanocomposite, Lac/Alg, to stabilize AgNPs without any additives, utilizing lactose as a reducing agent. The resulting nanocomposite AgNPs@Lac/Alg was used for the catalytic degradation of toxic dyes and investigated regarding its pH-dependent antibacterial activity.

## Results and Discussion

### Synthesis of AgNPs@Lac/Alg

**Figure 1:**
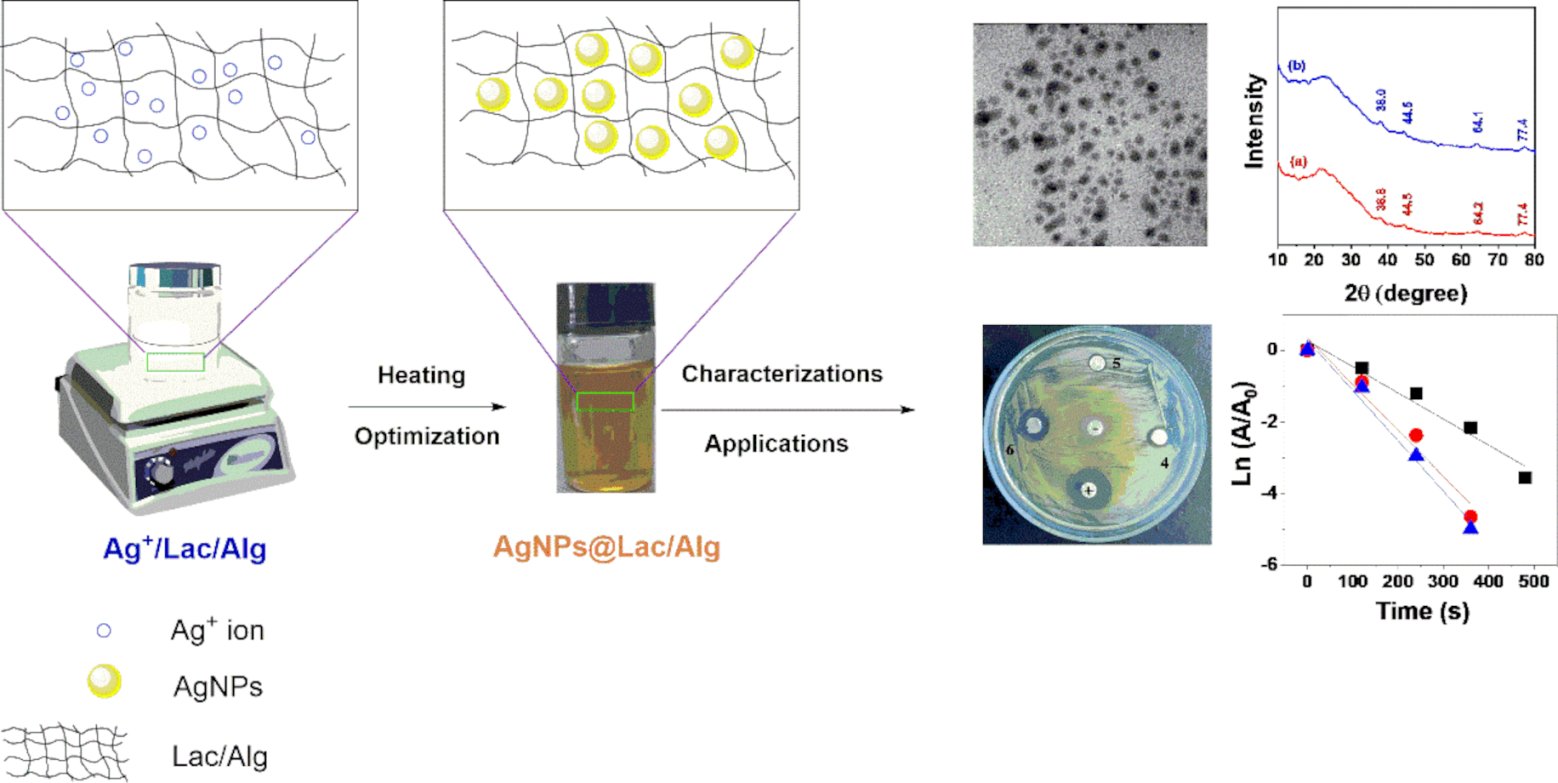
Schematic illustration for route study on AgNPs@Lac/Alg.

Our recent work has demonstrated the successful synthesis of AgNPs using a two-step process involving loading and reduction, resulting in nanocomposites composed of alginate and cyclodextrins [36,38]. In the first step, silver ions were loaded onto the nanocomposite [37,39], followed by reduction using plant extract [36,38]. In the present work, we have developed an in situ synthesis method for AgNPs using an alginate composite with lactose as reducing sugar. This method involves the use of lactose as a negatively charged compound that disrupts the cross-linking between Ca^2+^ ions and COO^−^ groups of sugar molecules in the alginate chains, leading to the formation of soluble nanoparticles (Figure 1).

To obtain the nanocomposite AgNPs@Lac/Alg, Ag^+^@Lac/Alg was reduced through a heating process, without the use of additional additives. The formation of AgNPs was confirmed by a change in the color of the colloidal solution. To find the optimal reaction conditions, the synthesis of the nanocomposite was carried out while varying the reaction conditions, such as temperature, reaction time, and ratio of Ag^+^ to blank composite. Subsequently, AgNPs@Lac/Alg was characterized using various analytical techniques and applied for the catalytic degradation of contaminants and in bacterial activity assays. Absorption spectra were analyzed to monitor changes in physicochemical properties at the maximum peak region of surface plasmon resonance (SPR) of the AgNPs, which falls in the range of 400–450 nm.

**Figure 2:**
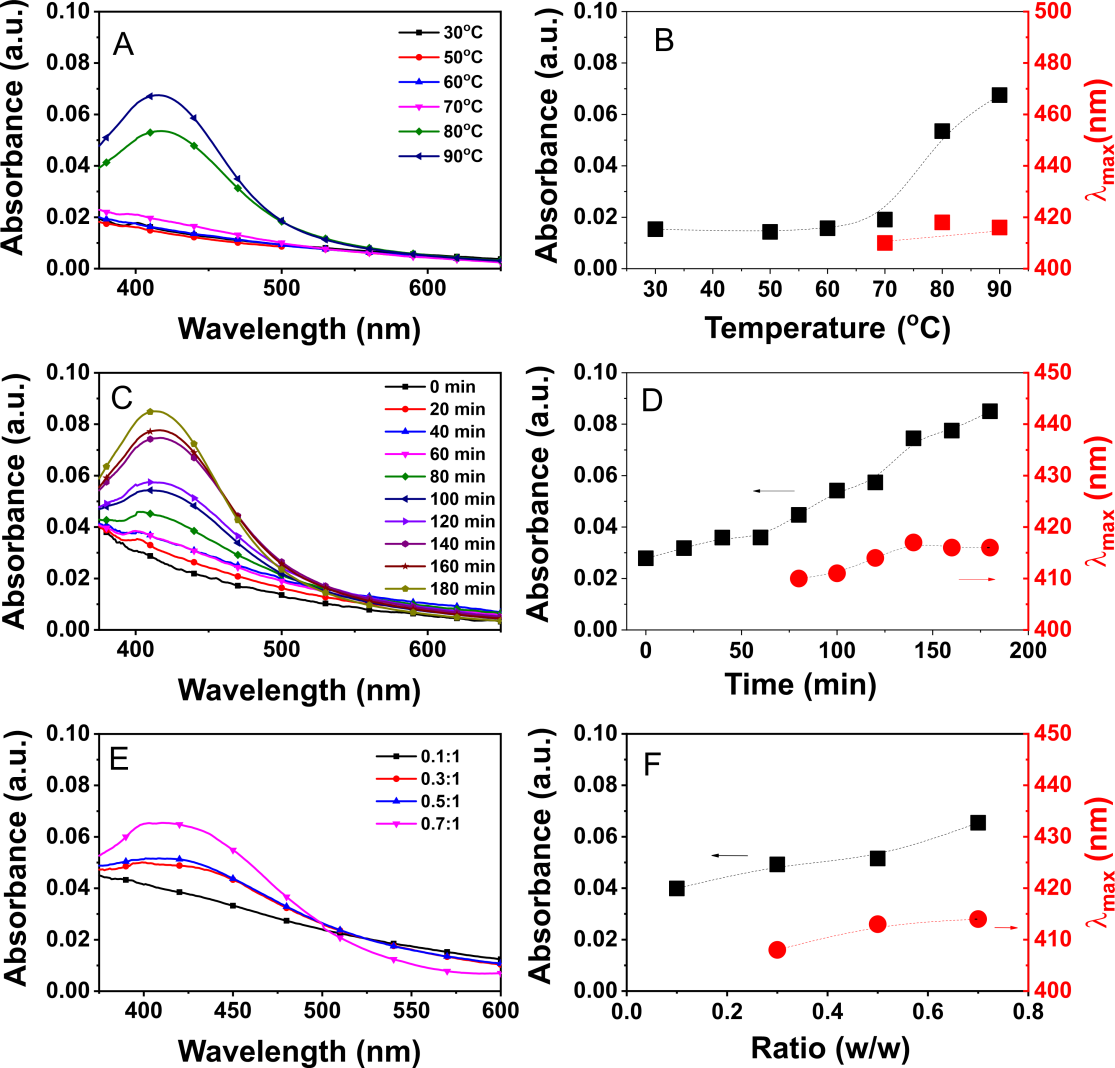
UV–vis spectra of AgNPs@Lac/Alg (left) and plots of absorbance and wavelength (λ_max_) data (right) recorded as a function of temperature (A and B), reaction time (C and D) and the ratio of silver ion to blank Lac/Alg (E and F).

The synthesis of stable AgNPs was performed at solution temperatures ranging from 30 to 90 °C. The results are shown in Figure 2A and Figure 2B. Below 70 °C, the SPR peak in the UV–vis spectra disappeared, indicating that no AgNPs were formed at lower solution temperatures. Increasing the reaction temperature resulted in a high concentration of AgNPs in the colloidal solution, with the highest intensity observed at a temperature of 90 °C. However, the increase in temperature did not significantly affect the morphology of the formed AgNPs, as evidenced by the absence of any notable change in λ_max_ values.

To investigate the influence of the reaction time, the gel Ag^+^@Lac/Alg was heated at a solution temperature of 90 °C and the UV–vis spectra were measured every 20 min. As seen in Figure 2C and Figure 2D, the formation of AgNPs in the colloidal solution was significantly influenced by the reaction time. The SPR band of AgNPs was not clearly observed within the initial 60 min, indicating a slow formation of the nanocomposite. However, after this period, AgNPs were rapidly produced, leading to a substantial increase in the absorbance values up to 140 min. Throughout the synthesis process, the λ_max_ values showed a slight increase, which confirms the critical role of reaction time in the synthesis of stable nanocomposites.

To assess the impact of silver ion concentration, ratios of silver ions to gel in the range of 0.1–0.7 (w/w) were optimized at 90 °C and 140 min. As shown in Figure 2E and Figure 2F, the concentration of silver ions strongly influenced the formation of AgNPs. A clear absorption peak was not observed at a ratio of 0.1 (w/w), indicating no formation of AgNPs. However, the increase in silver ions resulted in an increase in the absorbance value in the UV–vis spectra. Minor changes in λ_max_ values were noted from the absorption data, indicating a slight influence of the silver-ion-to-nanocomposite ratio on the physicochemical properties of the synthesized AgNPs. Considering these findings, two ratios of silver ions to Lac/Alg, namely 0.3 (AgNPs@Lac/Alg-0.3) and 0.7 (AgNPs@Lac/Alg-0.7), were selected for further physicochemical characterizations.

### Physicochemical characterizations

**Figure 3:**
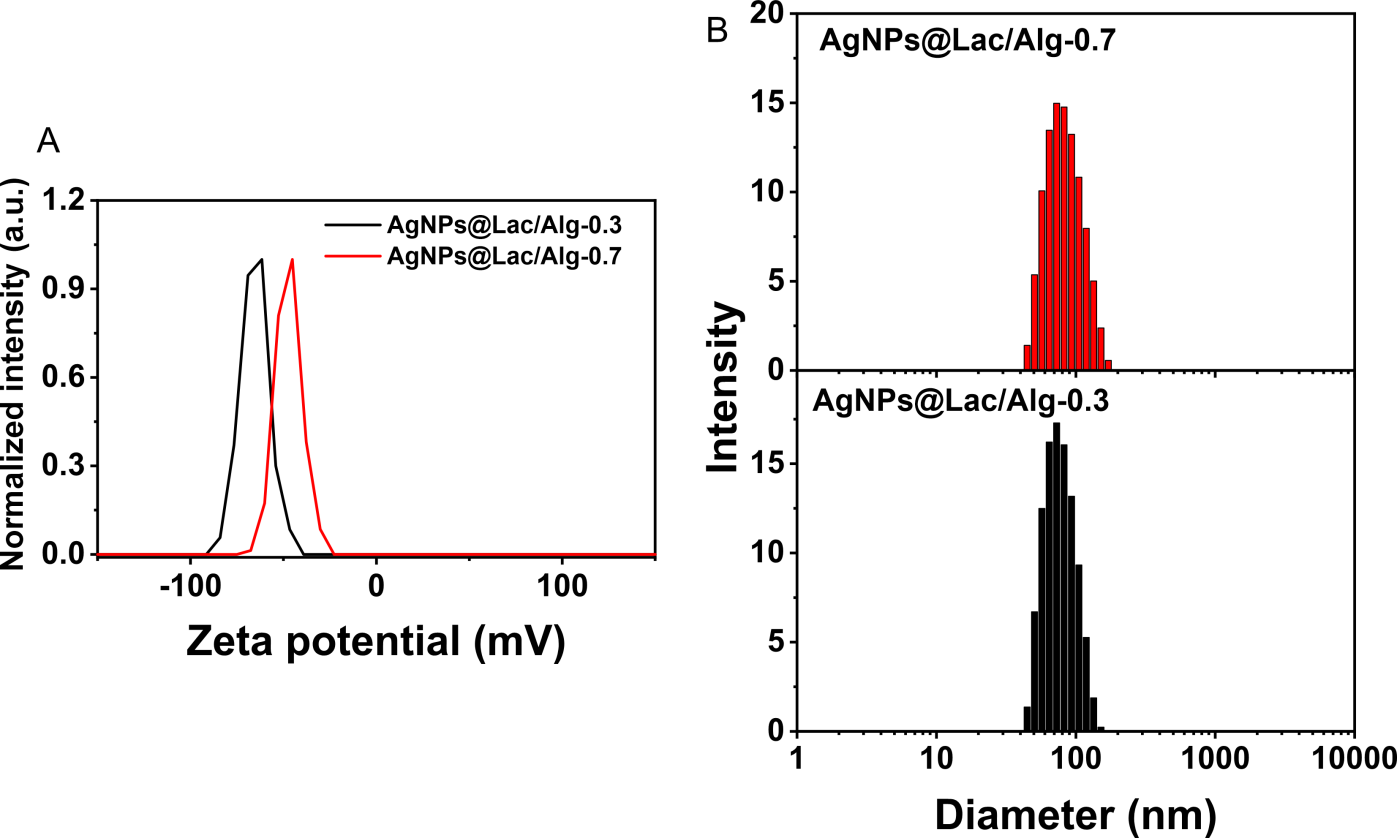
Zeta potentials (A) and size distribution obtained from DLS measurements (B) of AgNPs@Lac/Alg with different amount of AgNPs.

The stability and size of the nanocomposites in the colloidal solution were evaluated through zeta potential and DLS measurements at a temperature of 25 °C. The results are presented in Figure 3, demonstrating that all the synthesized nanocomposites were stable. The zeta potential values of AgNPs@Lac/Alg-0.3 and AgNPs@Lac/Alg-0.7 were −61.6 mV and −41.0 mV, respectively. These values were lower than that of the blank Lac/Alg nanocomposite (−102.7 mV) [37]. The decrease in zeta potential suggests that the presence of AgNPs reduced the negative charge of the nanocomposites, resulting in a decrease in their stability. The DLS data indicated that both nanocomposites exhibited wide size distributions ranging from 40–150 nm, with mean sizes of 73.9 ± 19.2 nm and 80.2 ± 24.1 nm for AgNPs@Lac/Alg-0.3 and AgNPs@Lac/Alg-0.7, respectively. These findings are consistent with a previous study on the synthesis of gold nanoparticles using this nanocomposite [37].

**Figure 4:**
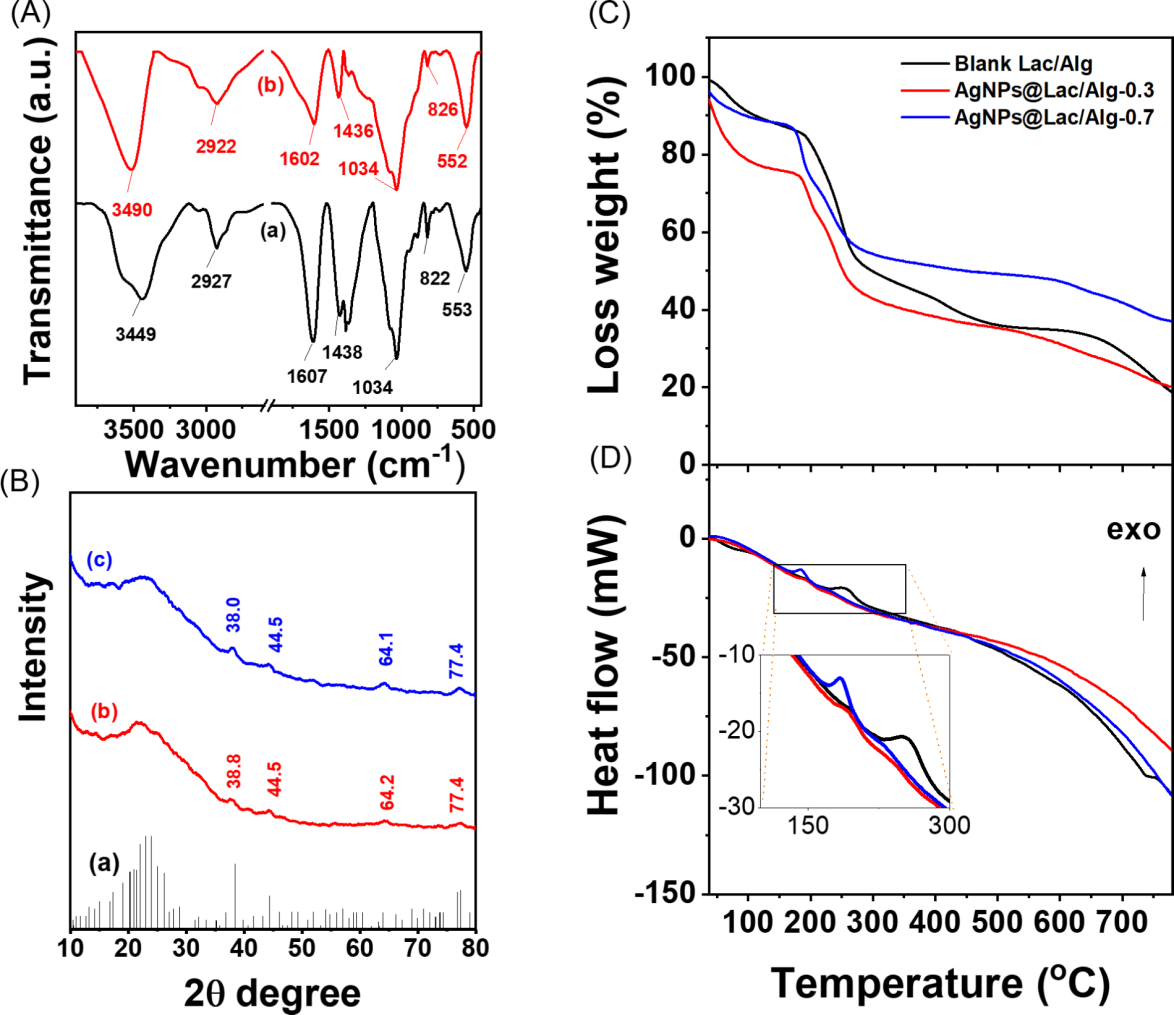
FTIR spectra (A) of AgNPs@Lac/Alg-0.3 (a) and AgNPs@Lac/Alg-0.7 (b); XRD patterns (B) of card number 00-004-0783 (a), AgNPs@Lac/Alg-0.3 (b), and AgNPs@Lac/Alg-0.7 (c); TGA (C) and DSC (D) curves of the different nanocomposites (the colors are the same as in panel (C)).

Figure 4 presents the FTIR spectra of the AgNPs@Lac/Alg nanocomposites. Both spectra exhibit similar signals, including broad band at around 3400 and 2900 cm^−1^, attributed, respectively, to the –OH and –CH stretching vibrations of the sugar units of polysaccharides [40]. Furthermore, single peaks at 1600 and 1436 cm^−1^ are observed, corresponding to the symmetric and asymmetric stretching vibrations of COO^−^ groups, respectively [41]. Additionally, the peaks at 1034 and 826 cm^−1^ are attributed to the stretching vibration of C–OH groups and the bending vibration of –CH groups [42]. Based on these data, it can be inferred that the primary components of the nanocomposites are polysaccharides.

To determine the crystalline structure of the nanocomposites, powder XRD patterns are evaluated and displayed in Figure 4B. Both nanocomposites exhibit feature peaks of AgNPs crystals at 2θ angles of about 38°, 44°, 64°, and 77°, indicating, respectively, the (111), (200), (220), and (311) planes of face-centered cubic silver (card number 00-004-0783). The XRD pattern of AgNPs@Lac/Alg-0.7 exhibits more intensive peaks compared to AgNPs@Lac/Alg-0.3, likely because of the different contents of AgNPs in the samples. The calculated XRD parameters are shown in Supporting Information File 1, Table S1. Using the Scherrer equation, the crystallite sizes of AgNPs in the nanocomposites were estimated to be 17.3 ± 2.7 nm for AgNPs@Lac/Alg-0.3 and 14.8 ± 2.5 nm for AgNPs@Lac/Alg-0.7. The mean lattice parameter and the cell volume of AgNPs in AgNPs@Lac/Alg-0.3 were determined to be 4.09 ± 0.02 Å and 68.5 ± 1.0 Å^3^, respectively, while those of AgNPs@Lac/Alg-0.7 were 4.09 ± 0.2 Å and 68.4 ± 0.8 Å^3^, respectively [43]. Thus, the XRD data provide evidence for the presence of AgNPs in the nanocomposites.

To evaluate the thermal properties of the AgNPs@Lac/Alg nanocomposites, TGA and DSC measurements were performed on both the blank composite and the nanocomposites in an air flow of 20 mL/min at a heating rate of 10 °C/min. The TGA curve of AgNPs@Lac/Alg closely resembles that of the blank composite. Between 37 and 200 °C, the nanocomposite AgNPs@Lac/Alg-0.3 exhibited a significantly higher thermal loss (28%) compared to the other compounds (ca. 10%), which can be attributed to the presence of hydrate and volatile components. In the range of 200–800 °C, the binding between the alginate chain and the AgNPs significantly stabilized the weight loss of Lac/Alg [37]. The weight losses of AgNPs@Lac/Alg-0.3 and AgNPs@Lac/Alg-0.7 were approximately 52 and 48%, respectively, while the blank nanocomposite underwent a weight loss of about 68%. The presence of similar quality of ashes remaining after the heating process confirms the presence of inorganic components in the nanocomposites that are comparable to those in the blank nanocomposite.

DSC analysis revealed weakly exothermic peaks in the range of 150–300 °C, corresponding to the oxidation of organic components in the nanocomposites. The temperature peak for the blank sample was found at 248 °C, while for the nanocomposites AgNPs@Lac/Alg-0.3 and AgNPs@Lac/Alg-0.7, the peaks occurred at 187 and 183 °C, respectively. These results suggest that the nanocomposites should be used in temperature regions below 200 °C.

**Figure 5:**
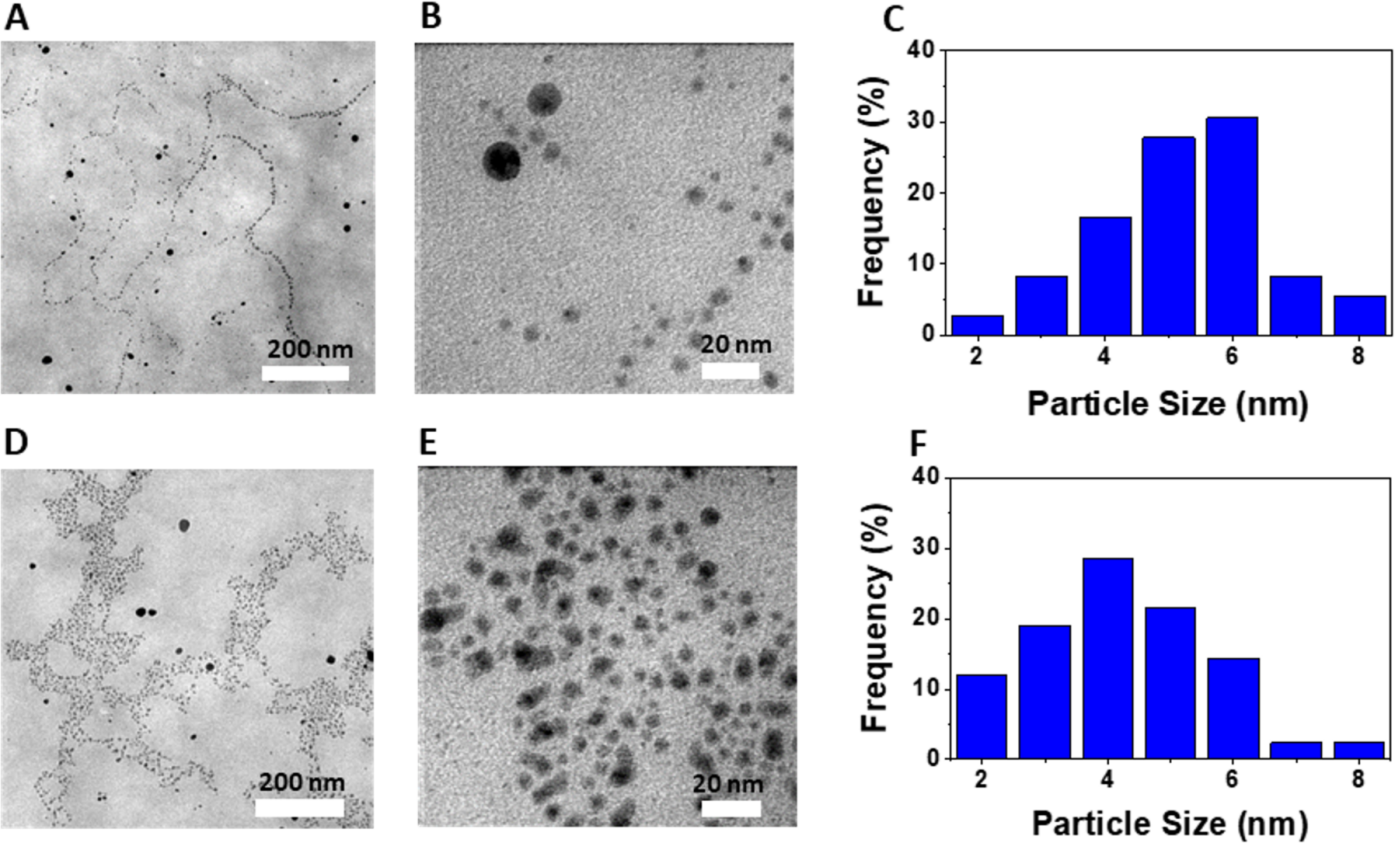
TEM images at different magnifications and particle size distributions of AgNPs@Lac/Alg-0.3 (A, B and C) and AgNPs@Lac/Alg-0.7 (D, E and F).

TEM images and size distributions of AgNPs@Lac/Alg are displayed in Figure 5. The TEM images demonstrate the presence of uniformly shaped spherical AgNPs. The size distribution analysis of AgNPs@Lac/Alg-0.3 and AgNPs@Lac/Alg-0.7 indicates that the highest frequency for AgNPs@Lac/Alg-0.3 is at 6 nm while for AgNPs@Lac/Alg-0.7, it is at 4 nm, with sizes ranging from 2 to 8 nm. The results show the similarity in physicochemical properties of the AgNPs@Lac/Alg nanocomposites. Thus, AgNPs@Lac/Alg-0.7 was selected for assessment of its catalytic and antibacterial properties in this study.

### Catalytic degradation of contaminants

**Figure 6:**
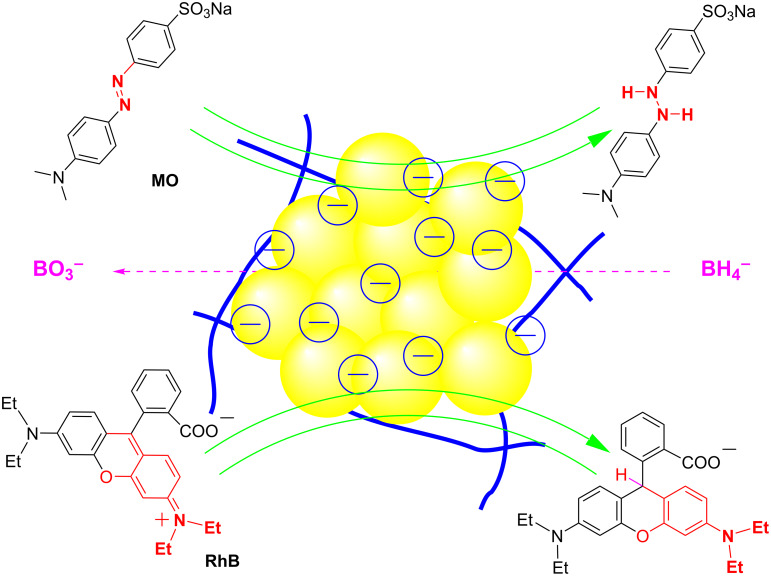
Proposed mechanism of AgNPs@Lac/Alg catalysis in reduction of methyl orange and rhodamine B using NaBH_4_.

Toxic organic dyes pose a serious threat to the environment, and their non-biodegradability in aqueous media has led to the consideration of various methods for their treatment [44–45]. In recent years, catalytic degradation using oxidizing and reducing agents such as H_2_O_2_ and NaBH_4_ has been frequently reported [46–48]. Plasmonic nanoscale metal particles have demonstrated enhanced catalytic performance in conjunction with NaBH_4_. The surface of the nanoscale metal particles served as an absorbent of dyes and BH_4_^−^ ions. Subsequently, an electron transfer process occurs from BH_4_^−^ (electron donor) to the dyes (electron acceptor) (Figure 6). As a result, the catalytic efficacy of metal NPs is significantly influenced by factors such as the surface characteristics and concentration of nanocatalyst, which directly impact the binding/adsorption capability of the reactant and the release/desorption of the reaction products [49]. This study aims to investigate the catalytic activity of the nanocomposite AgNPs@Lac/Alg for the degradation of two toxic dyes, methyl orange (MO) and rhodamine B (RhB), using various doses of the nanocomposite.

**Figure 7:**
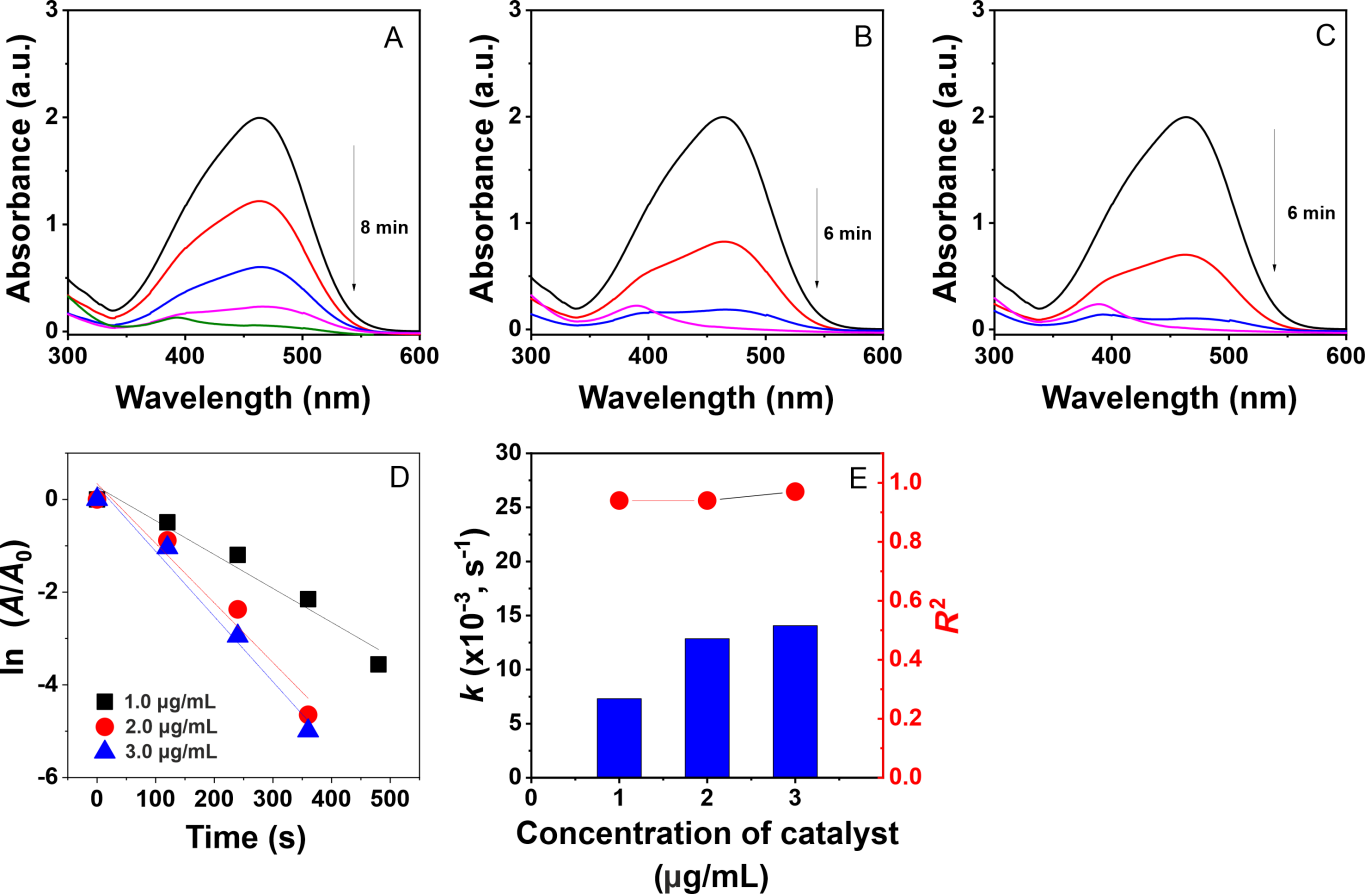
UV–vis spectra of the catalyst at concentrations of 1.0 μg/mL (A), 2.0 μg/mL (B), and 3.0 μg/mL (C); plots of first-order kinetics (D) and rate constants (E) for the catalytic degradation of methyl orange.

**Figure 8:**
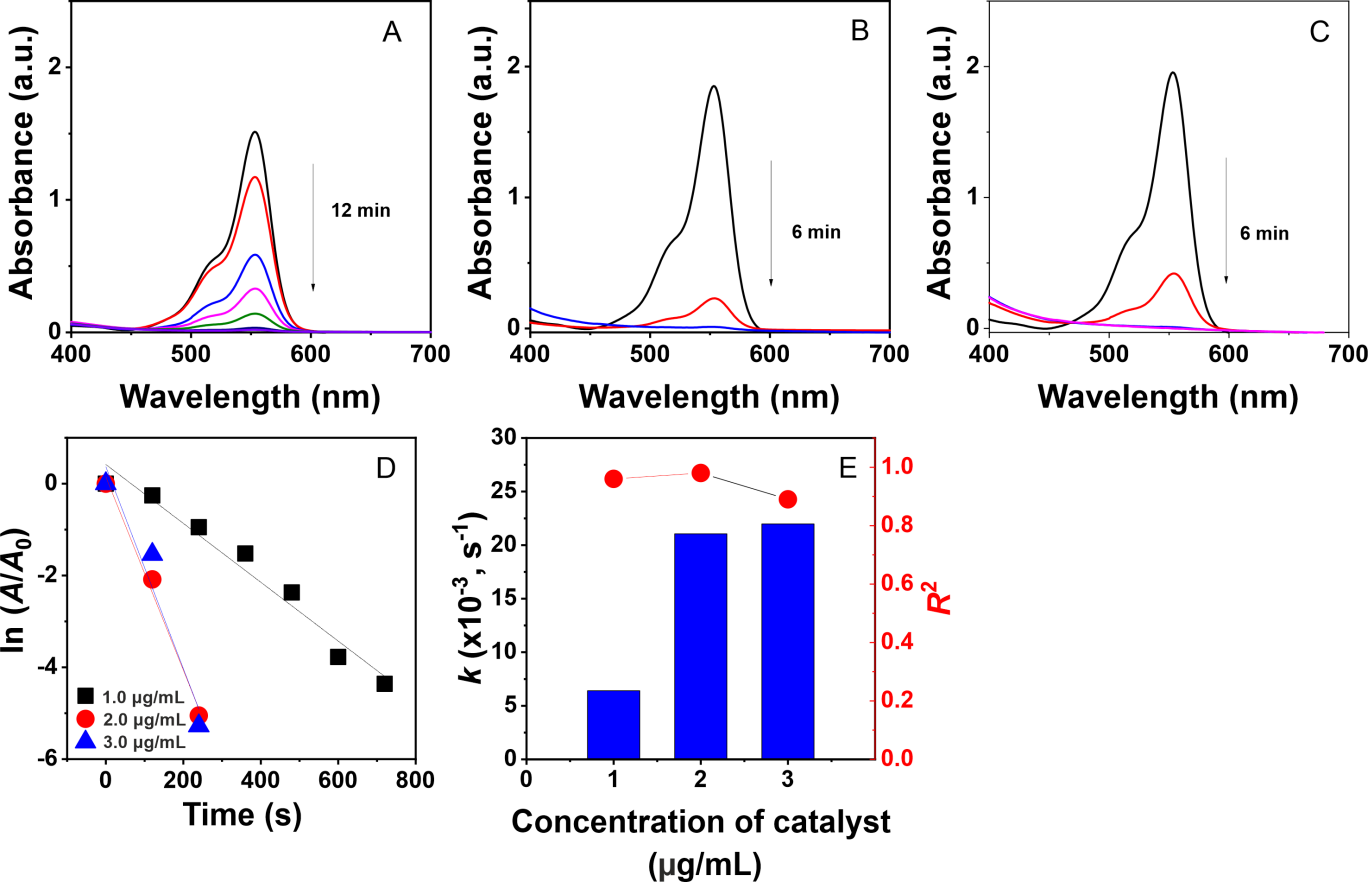
UV–vis spectra of the catalyst at concentrations of 1.0 μg/mL (A), 2.0 μg/mL (B), and 3.0 μg/mL (C); plots of first order kinetic (D) and rate constants (E) for the catalytic degradation of rhodamine B.

The degradation of toxic dyes using a nanocomposite catalyst was monitored in situ by measuring UV–vis absorption at ambient temperature, with the concentration changing in a time-dependent manner. The effectiveness of the reactions is confirmed by discoloration of the pollutant solution and a reduction in absorbance values in the UV–vis spectra. In a separate study [45], it was shown that the dyes degraded weakly in the absence of a catalyst. As seen in Figure 7 and Figure 8, the reduction of MO and RhB is achieved using different doses of the AgNPs@Lac/Alg solution (1.0–3.0 μg/mL), resulting in the reduction of the absorption peaks at 467 and 553 nm, respectively. The reaction rate increases with the catalyst dose as evidenced by a decrease in the reaction time. For instance, the degradation of MO at a dose of the colloidal solution of 1.0 μg/mL was completed in 8 min, while it took only 6 min in the presence of 2.0 μg/mL and 3.0 μg/mL of the nanocatalyst. The addition of the nanocatalyst also facilitated the degradation of RhB, with complete reaction times of 12 min for 1.0 μg/mL and 6 min for 2.0 μg/mL and 3.0 μg/mL of the nanocatalyst.

For the reaction kinetics, the plots of ln(*A*_0_/*A*_t_) values versus reaction time show good linear correlation with high determination coefficients for the degradation of the organic dyes, indicating a pseudo-first-order reaction (Figure 7E and Figure 8E). Furthermore, the calculated rate constants increase with higher concentrations of the nanocatalyst. Specifically, the rate constant for MO degradation increases from 7.31 × 10^−3^ s^−1^ (*R*^2^ = 0.94) for 1.0 μg/mL of AgNPs@Lac/Alg to 14.07 × 10^−3^ s^−1^ (*R*^2^ = 0.97) for 3.0 μg/mL of AgNPs@Lac/Alg. For RhB degradation, the calculated values at 1.0 μg/mL, 2.0 μg/mL, and 3.0 μg/mL of AgNPs@Lac/Alg are 6.41 × 10^−3^ s^−1^ (*R*^2^ = 0.96), 21.05 × 10^−3^ s^−1^ (*R*^2^ = 0.98), and 21.98 × 10^−3^ s^−1^ (*R*^2^ = 0.89), respectively. The rate constants of AgNPs@Lac/Alg are found to be higher than the values of metal NPs loaded on alginate-based nanocomposites used in the solid state [36–38]. These results suggest that amount and carrier property of the metal NPs play a significant role in the catalytic degradation of organic dyes.

### Antibacterial activity

**Figure 9:**
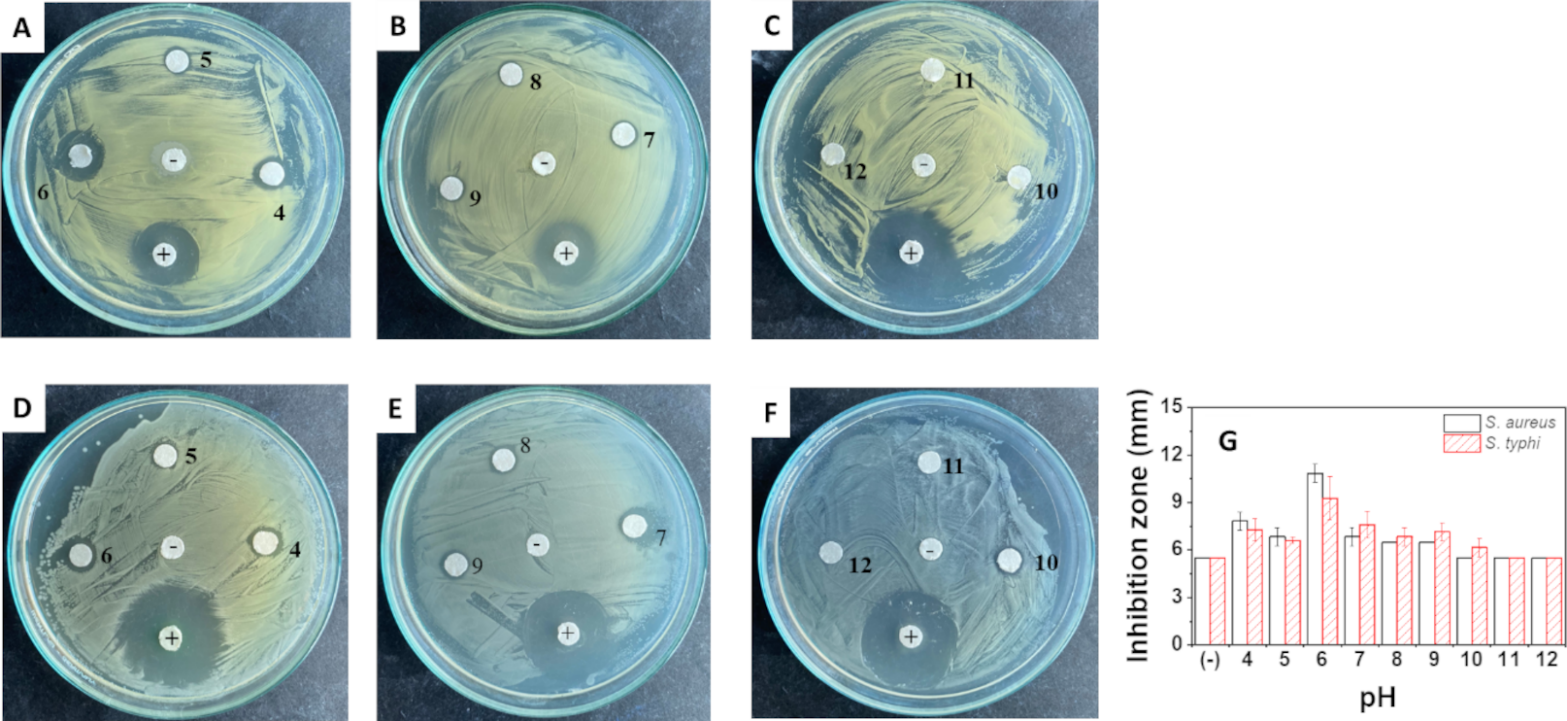
Antibacterial activity of AgNPs@Lac/Alg tested with various pH (4–12) against *S. aureus* (A, B and C) and *S. typhi* (D, E and F) and inhibition zone diameters as functions of the pH value (G).

The effectiveness of AgNPs@Lac/Alg in inhibiting the growth of bacteria was tested on the two microbial strains *S. aureus* (Gram-positive) and *S. typhi* (Gram-negative) using the disk diffusion method at different pH values. To establish the bioactivity of the nanocomposite, antibacterial assays were also conducted in media with different pH values without the nanocomposite. These resulted in no bioactivity against either of the strains, indicating that the observed bioactivity was due to the nanocomposite alone (Supporting Information File 1, Figure S1). Inhibition images and inhibition zone plots of AgNPs@Lac/Alg at different pH values are presented in Figure 9. The results show that the antibacterial activity of the nanocomposite is pH-dependent, and the ability to inhibit bacterial growth can be attributed to the leaching of silver (ions or nanoparticles) from the nanocomposite into the medium. The nanocomposite exhibited the highest antibacterial activity at weakly acidic and basic pH values, with the highest inhibition zone diameters recorded at pH 6 against *S. aureus* (10.8 ± 0.57 mm) and *S. typhi* (9.3 ± 1.34 mm). In contrast, the nanocomposite showed no inhibition in highly basic media (pH 11 and 12) because of the aggregation of nanoparticles, which hindered the leaching of silver into the medium. The antibacterial effectiveness of the nanocomposite varied among the bacterial strains and pH values, which may be related to the antibacterial mechanism of AgNPs and the cell wall and membrane structure of each bacterial strain [50]. These findings suggest that the antibacterial activity of the nanocomposite could be tailored to specific pharmaceutical applications, such as drug delivery, by adjusting the release medium.

**Figure 10:**
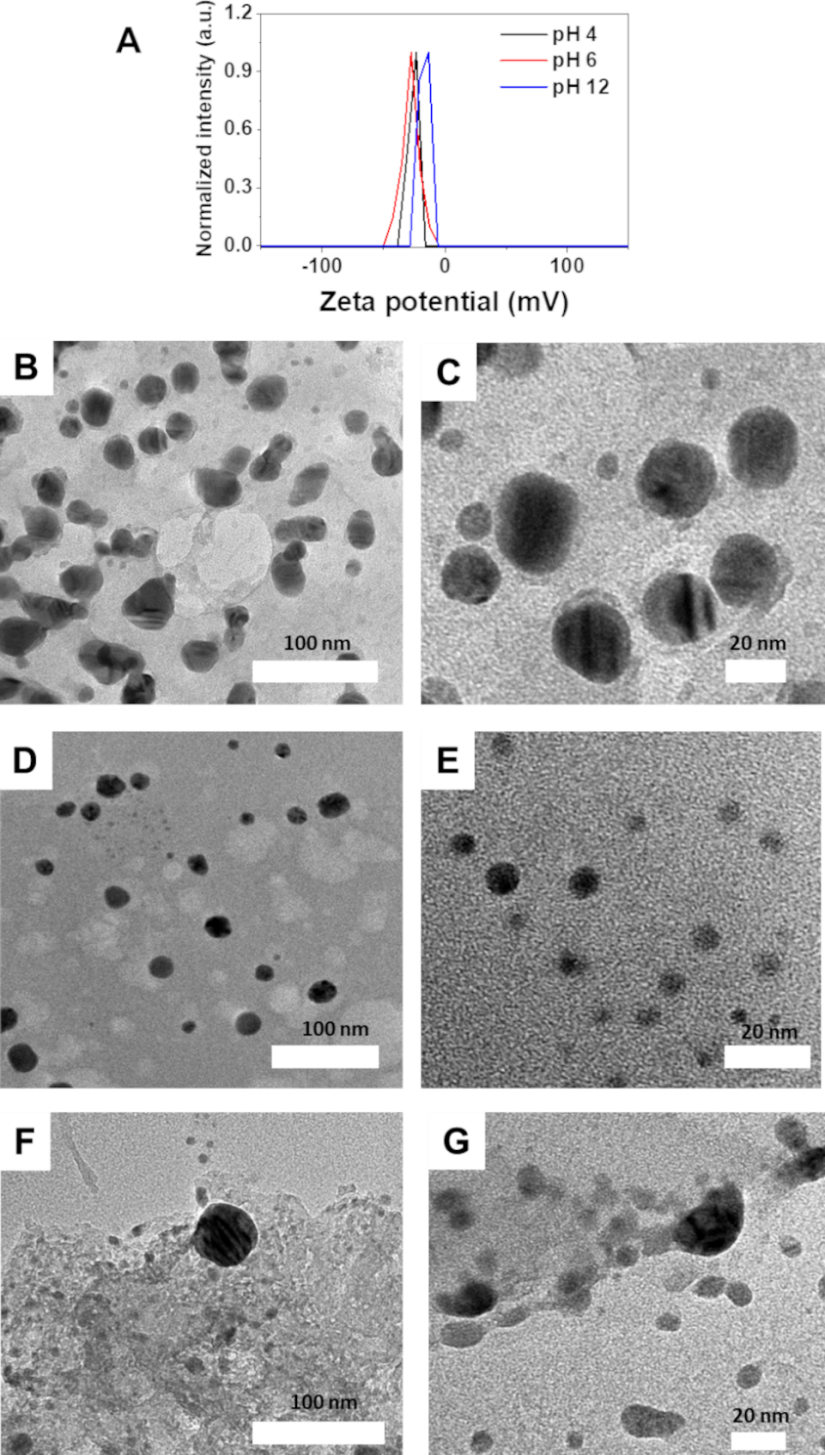
Zeta potential (A) and TEM images of the nanocomposite solutions at pH 4 (B and C), pH 6 (D and E) and pH 12 (F and G).

To investigate the pH-dependent antibacterial effectiveness, zeta potential and morphology of the nanocomposite were examined at pH 4, 6, and 12, as shown in Figure 10. The zeta potentials of the nanocomposite were −23.3, −27.5, and −13.3 mV at pH 4, 6, and 12, respectively, indicating that the stability of the nanocomposite was low in highly acidic and basic media, leading to the aggregation of nanoparticles. TEM images demonstrated significant variations in the morphology of the nanocomposite in different media. At pH 4, AgNPs aggregated to form larger particles compared to those formed at pH 6. In contrast, at pH 12, the nanocomposite was disrupted, leading to the aggregation of gel and a reduced interaction between AgNPs and the bacterial cell wall. This accounts for the low activity of the nanocomposite in highly basic media.

## Conclusion

A lactose/alginate nanocomposite was successfully employed for the in situ synthesis of AgNPs. This study presents a novel polysaccharide-based nanocomposite with a straightforward synthesis approach. The AgNPs@Lac/Alg synthesized under optimal conditions had an average size in the range of 4–6 nm. Remarkable catalytic performance was demonstrated regarding the degradation of two contaminants. The antibacterial activity of the nanocomposite was found to be pH-dependent, which can be attributed to the varying surface properties of silver nanoparticles. The highest bioactivity was observed at pH 6. These findings suggest that the nanocomposite may be customized for specific applications in environmental and medicinal treatments, making it a promising material.

## Experimental

### Materials

The following chemicals and reagents were purchased from Acros (Belgium): silver nitrate (AgNO_3_), methyl orange (MO), rhodamine B (RhB), calcium acetate hydrate, sodium alginate, and sodium tetrahydroborate (NaBH_4_). Lactose was obtained from Yong Da (China). The chemicals were used without additional purification. Distilled water was utilized throughout the experiments.

### Synthesis of AgNPs@Lac/Alg

The AgNPs@Lac/Alg composite was produced by in situ reduction of silver ions to form AgNPs with the aid of the Lac/Alg nanocomposite. The Lac/Alg nanocomposite was synthesized using an ionic gelation method as previously reported [37]. Briefly, an aqueous solution of calcium acetate (14.36 mL; 6.5 mg·mL^−1^) was slowly added dropwise into an aqueous solution of sodium alginate (40 mL; 7 mg·mL^−1^) under stirring for 1 h at 1200 rpm. The mixture was allowed to equilibrate overnight. Subsequently, the Alg/Ca^2+^ spheres were separated by centrifugation at 3000 rpm for 10 min and washed three times with distilled water (20 mL each) to remove impurities. Next, an aqueous solution of lactose (16.06 mL, 1.5 mg·mL^−1^) was added to the Alg/Ca^2+^ spheres and stirred for 1 h at 1200 rpm. To ensure the formation of a stable gel, the Lac/Alg gel solution was ultrasonically treated for 30 min and left to reach equilibrium overnight. After purification, the resulting gel was freeze-dried at −80 °C for 24 h using an EYELA FDU-2110 (Japan) instrument. Next, an aqueous solution of AgNO_3_ (15 mL) with different concentrations in the range of 7.9–55.6 μg·mL^−1^ was mixed with 3 mL of an aqueous solution containing Lac/Alg nanocomposite (4.37 mg·mL^−1^) and stirred for 1 h. The mixture was heated, and the formation of AgNPs was confirmed by a change in color of the mixture. The influence of the reaction parameters, namely temperature (30–90 °C), reaction time (0–180 min), and weight ratio of silver to Lac/Alg composite (0.1:1 to 0.7:1 (w/w)), was scrutinized using UV–vis measurements (UV-5100, Shanghai Metash Intruments Co. Ltd) in the range of 350–600 nm. For investigating the influence of temperature, a ratio of silver to Lac/Alg composite of 0.6:1 was used for a reaction time of 120 min. The influence of the reaction time was investigated with a ratio of silver to Lac/Alg composite of 0.6:1 and a solution temperature of 90 °C. The optimal ratio of silver to Lac/Alg composite was determined in reactions at 90 °C for 120 min. The AgNPs@Lac/Alg nanocomposite was then centrifuged and washed with distilled water. The purified nanocomposite was stored at 4 °C for further experiments.

### Characterizations of AgNPs@Lac/Alg nanocomposite

The size distribution and stability of the nanocomposites were determined using dynamic light scattering (DLS) and zeta potential measurements, which were measured on a nanoPartica Horiba SZ-100 (Japan). Fourier-transform infrared (FTIR) spectra were obtained using a Bruker Tensor 27 FTIR spectrophotometer (Germany). X-ray diffraction (XRD) patterns were collected using a Bruker D8 Advance X-ray diffractometer. The morphology of the nanocomposites was investigated using transmission electron microscopy (TEM) with a JEOL JEM-1400 instrument. Thermal analysis, including thermogravimetry analysis (TGA) and differential scanning calorimetry (DSC), was conducted using a LabSys Evo 1600 thermal analyzer (SETARAM, France) under atmospheric conditions from 25 to 800 °C with a heating rate of 10 °C/min.

### Catalytic activity for degradation of contaminants

Catalytic activity of the synthetic nanocomposite was evaluated through the degradation of pollutants (MO and RhB) in the presence of excess amounts of NaBH_4_ [51]. In a quartz cell with a path length of 1 cm, 2.5 mL of a solution containing 0.1 mM of the contaminants and an aqueous nanocomposite solution of catalyst (AgNPs@Lac/Alg-0.7, in which the ratio of Ag^+^ to Lac/Alg was 0.7:1, w/w) at concentrations ranging from 1.0 to 3.0 μg/mL were added. Subsequently, 0.5 mL of NaBH_4_ solution (0.05 M) was introduced into the cell. Using a UV–vis spectrophotometer, the catalytic degradation of the contaminants was monitored at ambient temperature in 2 min intervals. The reaction kinetics were determined by measuring the reduction in absorption intensity at the corresponding absorption peak. It is important to note that the catalytic degradation of these contaminants in the presence of excess amount of NaBH_4_ is a pseudo-first-order reaction, which can be expressed by the equation ln(*A*/*A*_0_) = −*kt*. Here, *k* is reaction rate constant, and [*A*_0_] and [*A*] are initial and current absorbance of the contaminant, respectively [43]. The catalytic efficiency of the nanocomposite was determined by calculating the rate constant from the slope of the straight line obtained from plots of ln(*A*/*A*_0_) versus reaction time.

### Effect of pH value and antibacterial assay

The impact of pH value on the stability of the nanocomposite was analyzed under various acidic and alkaline conditions within the pH range of 4 to 12. An aqueous solution of the nanocomposite AgNPs@Lac/Alg-0.7 (1 mL) was added to HCl or NaOH solutions, resulting in 3 mL solution with the corresponding pH values. The solutions were used to experiment antibacterial activity. The solutions with pH 4, 6, and 12 were also used to determine stability of the nanocomposite through zeta potential measurements and TEM.

The disk diffusion method was employed to assay the antibacterial activity of the samples [52]. In order to conduct the antibacterial tests, the nanocomposite solution (0.3 mL) at various pH values was diluted in DMSO (10 mL) to obtain a composite solution (0.87 μg/mL) prior to the experiment. *Staphylococcus aureus* (a Gram-positive bacterium) and *Salmonella typhi* (a Gram-negative bacterium) were used for the tests. Each aliquot (20 μL) was placed on paper disks with a diameter of 5.5 mm. These disks were then placed on agar plates that were previously inoculated with bacterial cultures (1.0 mL, 10^6^ CFU/mL). The antibiotic ampicillin (0.01 mg/mL) and Luria Bertani broth were utilized as positive and negative controls, respectively. The same protocol was applied to evaluate media with the corresponding pH values and without the nanocomposite. The inhibition zone diameter including the paper disk, was measured to determine the activity.

## Supporting Information

Supporting Information features physical parameters calculated from XRD patterns and antibacterial activity of controls without nanocomposite.

File 1 Additional experimental data.
