# Supplementary material for: Silver nanoparticles loaded on lactose/alginate: in situ synthesis, catalytic degradation, and pH-dependent antibacterial activity
Source: Beilstein J Nanotechnol. 2023 Jul 4;14:781–92. doi: 10.3762/bjnano.14.64 (PMC10334209; doi:10.3762/bjnano.14.64)
Supplement: File 1 — Additional experimental data. [file Beilstein_J_Nanotechnol-14-781-s001.pdf]

BEILSTEIN JOURNAL OF NANOTECHNOLOGY

Supporting Information

for

Silver nanoparticles loaded on lactose/alginate: in situ synthesis, catalytic degradation, and pH-dependent antibacterial activity

Nguyen Thi Thanh Tu, T. Lan-Anh Vo, T. Thu-Trang Ho, Kim-Phuong T. Dang, Van-Dung Le, Phan Nhat Minh, Chi-Hien Dang, Vinh-Thien Tran, Van-Su Dang, Tran Thi Kim Chi, Hieu Vu-Quang, Radek Fajgar, Thi-Lan-Huong Nguyen, Van-Dat Doan and Thanh-Danh Nguyen

Beilstein J. Nanotechnol. **2023,** *14,* 781–792. doi:10.3762/bjnano.14.64

Additional experimental data

License and Terms: This is a supporting information file under the terms of the Creative Commons Attribution License (https://creativecommons.org/licenses/by/4.0). Please note that the reuse, redistribution and reproduction in particular requires that the author(s) and source are credited and that individual graphics may be subject to special legal provisions.
The license is subject to the *Beilstein Journal of Nanotechnology* terms and conditions: (https://www.beilstein-journals.org/bjnano/terms)

Table S1: Physical parameters calculated from XRD patterns.

**Materials**	**No.**	**2θ (degree)**	**cos θ**	**FWHM (Degree)**	**β (radian)**	**Crystallite size 'D' (μm)**	**Interplanar spacing *d* (Å)**	***h k l* Identified from peak**	**$h^2+k^2+l^2$**	**Lattice const. *a* from *d* (Å)**	**Cell volume (Å³)**
**AgNPs@Lac/Alg-0.3**	1	38.8347	0.3389	0.9431	0.48	0.008378	17.5487	2.3759	1 1 1	3	4.1152
	2	44.5260	0.3886	0.9254	0.41	0.007156	20.9371	2.0332	2 0 0	4	4.0664
	3	64.2130	0.5604	0.8470	0.62	0.010821	15.1268	1.4493	2 2 0	8	4.0993
	4	77.4092	0.6755	0.7803	0.66	0.011519	15.4243	1.2319	3 1 1	11	4.0857
**AgNPs@Lac/Alg-0.7**	1	38.0153	0.3317	0.9454	0.48	0.008377	17.5050	2.3651	1 1 1	3	4.0965
	2	44.5010	0.3883	0.9255	0.54	0.009424	15.8952	2.0343	2 0 0	4	4.0686
	3	64.1030	0.5594	0.8476	0.80	0.013962	11.7162	1.4515	2 2 0	8	4.1055
	4	77.3992	0.6754	0.7804	0.72	0.012566	14.1379	1.2320	3 1 1	11	4.0861

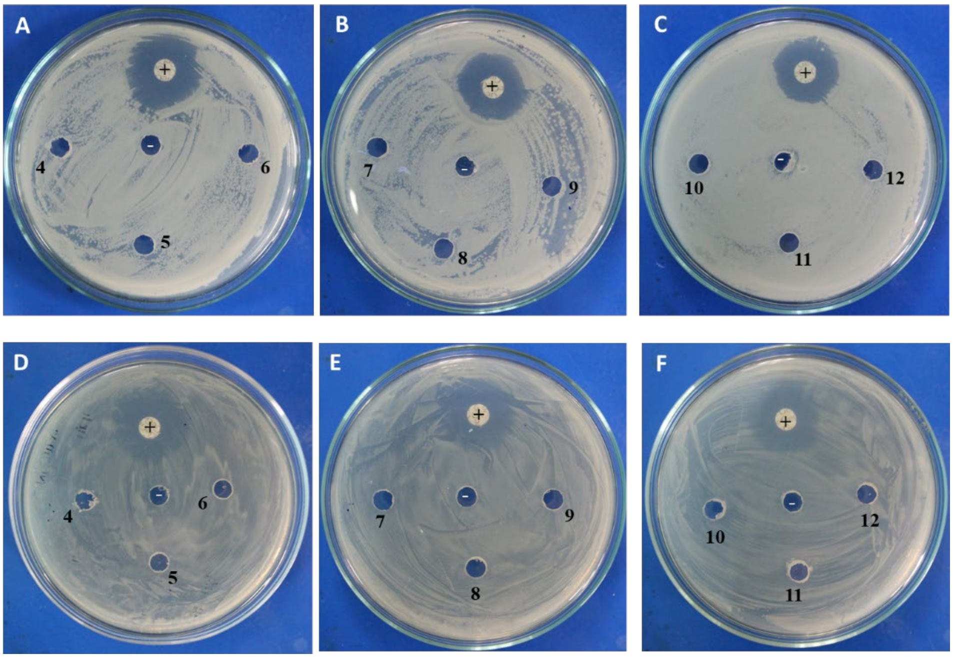

Figure S1: Antibacterial activity of controls with indicated pH value without nanocomposite. The controls exhibit no inhibition against both bacterial strains.
